# Open-label clinical trial of bezafibrate treatment in patients with fatty acid oxidation disorders in Japan

**DOI:** 10.1016/j.ymgmr.2018.02.003

**Published:** 2018-02-22

**Authors:** Kenji Yamada, Hideaki Shiraishi, Eishin Oki, Mika Ishige, Toshiyuki Fukao, Yusuke Hamada, Norio Sakai, Fumihiro Ochi, Asami Watanabe, Sanae Kawakami, Kazuyo Kuzume, Kenji Watanabe, Koji Sameshima, Kiyotaka Nakamagoe, Akira Tamaoka, Naoko Asahina, Saki Yokoshiki, Takashi Miyakoshi, Kota Ono, Koji Oba, Toshiyuki Isoe, Hiroshi Hayashi, Seiji Yamaguchi, Norihiro Sato

**Affiliations:** a Department of Pediatrics, Shimane University Faculty of Medicine, 89-1, En-ya-cho, Izumo, Shimane 693-8501, Japan; b Department of Pediatrics, Hokkaido University School of Medicine, Kita 15, Nishi 7, Kita-ku, Sapporo 060-8638, Japan; c Department of Pediatrics, Tsugaru General Hospital, 12-3, Iwaki-cho, Goshogawara, Aomori 037-0074, Japan; d Department of Pediatrics and Child Health, Nihon University School of Medicine, 1-6, Kanda-Surugadai, Chiyoda-ku, Tokyo 101-8309, Japan; e Department of Pediatrics, Graduate School of Medicine, Gifu University, 1-1, Yanagito, Gifu 501-1194, Japan; f Department of Pediatrics, Osaka University Faculty of Medicine, 2-2, Yamadaoka, Suita, Osaka 565-0871, Japan; g Department of Pediatrics, Osaka Hospital, Japan Community Healthcare Organization, 4-2-78, Fukushima, Fukushima-ku, Osaka 553-0003, Japan; h Department of Pediatrics, Yawatahama City General Hospital, 638, Ohira-ichibankochi, Yawatahama, Ehime 796-8502, Japan; i Department of Pediatrics, Ehime University Graduate School of Medicine, Shitsukawa, Toon, Ehime 791-0295, Japan; j Department of Community and Emergency Medicine, Ehime University School of Medicine, Shitsukawa, Toon, Ehime 791-0295, Japan; k Department of Pediatrics, Kagoshima City Hospital, 37-1, Uearata-cho, Kagoshima 890-8760, Japan; l Department of Neurology, Division of Clinical Medicine, Faculty of Medicine, University of Tsukuba, 1-1-1, Tennoudai, Tsukuba, Ibaraki 305-8575, Japan; m Hokkaido University Hospital Clinical Research and Medical Innovation Center, Research and Development Division, Kita 14, Nishi 5, Kita-ku, Sapporo 060-8648, Japan; n Hokkaido University Hospital Clinical Research and Medical Innovation Center, Biostatistics Division, Kita 14, Nishi 5, Kita-ku, Sapporo 060-8648, Japan; o Department of Biostatistics, School of Public Health, Graduate School of Medicine, The University of Tokyo, 7-3-1, Hongo, Bunkyo-ku, Tokyo 113-0033, Japan; p Hokkaido University Hospital Clinical Research and Medical Innovation Center, Kita 14, Nishi 5, Kita-ku, Sapporo 060-8648, Japan

**Keywords:** Bezafibrate, Fatty acid oxidation disorders (FAODs), Very long-chain acyl-CoA dehydrogenase (VLCAD) deficiency, Carnitine palmitoyltransferase-II (CPT-2) deficiency, Clinical trial

## Abstract

**Introduction:**

Fatty acid oxidation disorders (FAODs) are rare diseases caused by defects in mitochondrial fatty acid oxidation (FAO) enzymes. While the efficacy of bezafibrate, a peroxisome proliferator-activated receptor agonist, on the *in vitro* FAO capacity has been reported, the *in vivo* efficacy remains controversial. Therefore, we conducted a clinical trial of bezafibrate in Japanese patients with FAODs.

**Materials and methods:**

This trial was an open-label, non-randomized, and multicenter study of bezafibrate treatment in 6 patients with very long-chain acyl-CoA dehydrogenase (VLCAD) deficiency and 2 patients with carnitine palmitoyltransferase-II (CPT-2) deficiency (median age, 8.2 years; ranging from 5.8 to 26.4 years). Bezafibrate was administered for 6 months following a 6-month observation period. The primary endpoint was the frequency of myopathic attacks, and the secondary endpoints were serum acylcarnitines (ACs, C14:1 or C16 + C18:1), creatine kinase (CK) levels, degree of muscle pain (VAS; visual analog scale) during myopathic attacks, and quality of life (QOL; evaluated using validated questionnaires).

**Results:**

The frequency of myopathic attacks after bezafibrate administration decreased in 3 patients, increased in 3, and did not change in 2. The CK, AC, and VAS values during attacks could be estimated in only three or four patients, but a half of the patients did not experience attacks before or after treatment. Changes in CK, AC, and VAS values varied across individuals. In contrast, three components of QOL, namely, physical functioning, role limitation due to physical problems (role physical), and social functioning, were significantly elevated. No adverse drug reactions were observed.

**Conclusion:**

In this study, the frequency of myopathic attacks and CK, AC, and VAS values during the attacks could not be evaluated due to several limitations, such as a small trial population. Our findings indicate that bezafibrate improves the QOL of patients with FAODs, but its efficacy must be examined in future investigations.

## 1 Introduction

Mitochondrial fatty acid oxidation disorders (FAODs) are caused by defects in mitochondrial enzymes involved in fatty acid β-oxidation (FAO), which play an important role in energy production during periods when energy production from carbohydrates is reduced [1]. FAO enzymes include very long-, medium-, and short-chain acyl-CoA dehydrogenases (VLCAD, MCAD, and SCAD, respectively), trifunctional protein (TFP), carnitine-acylcarnitine translocase (CACT), carnitine palmitoyltransferase-II (CPT-2), electron transfer flavoprotein, and electron transfer flavoprotein dehydrogenase. Patients with FAODs exhibit various symptoms triggered by infection, diarrhea, long fasting, or muscular exertion [2]. FAODs are clinically classified into 3 types: (1) neonatal onset type, which develops during the neonatal period or early infancy with profound hypoglycemia, hepatic dysfunction, or cardiac failure and is often fatal; (2) infantile onset type (intermediate type), which exhibits intermittent attacks of lethargy, hepatic dysfunction, hypoglycemia, and occasionally encephalopathy or even sudden infant death; and (3) adult onset myopathic type, which involves episodic attacks of muscle weakness, myalgia, myoglobinuria, or rhabdomyolysis after school age [3]. Although no drug treatments are currently available for FAODs, avoiding long fasting, minimizing exercise, maintaining a low fat and high carbohydrate diet, and using medium-chain triglyceride oil for long-chain FAODs in the stable condition are used for prophylaxis against metabolic attacks. Further, early glucose infusion is recommended in acute phases such as pyrexia, diarrhea, or lethargy [4].

Bezafibrate [2-(p-(2-(p-chlorobenzamido)ethyl)-phenoxy)-2-methyl propionic acid] is a peroxisome proliferator-activated receptor (PPAR) agonist [5] that decreases human serum lipid levels [6,7]. Recent reports demonstrated that bezafibrate may represent a promising drug for FAODs by enhancing the transcription of several β-oxidation enzymes *in vitro* [[8], [9], [10], [11]]. Moreover, the clinical efficacy of bezafibrate for CPT-2 or multiple acyl-CoA dehydrogenase (MAD) deficiencies was recently reported by French and Japanese groups, respectively [[12], [13], [14]]. Conversely, a 3-month, randomized, double-blind, crossover study involving a bezafibrate clinical trial in patients with CPT-2 and VLCAD deficiencies failed to demonstrate improvement in clinical symptoms or in some physical abilities [15]. Hence, the clinical efficacy of bezafibrate for FAODs is still controversial [[16], [17], [18]].

In this study, we evaluated the efficacy and safety of bezafibrate for treating patients with FAODs by a clinical trial resembling the study design of a previously reported French clinical trial [12].

## 2 Materials and methods

### 2.1 Design

This study was a non-randomized, uncontrolled multicenter, open-label trial.

### 2.2 Standard protocol approvals, registrations, and patient consents

This study was approved by the Institutional Review Boards (IRBs) of Hokkaido University Hospital and the other collaborating institutions. All patients were registered based on the database in the Department of Pediatrics, Shimane University Faculty of Medicine. Written informed consent for study participation was obtained from all patients or their parents.

### 2.3 Setting

The study was conducted at Hokkaido University Hospital Clinical Research and Medical Innovation Center, Japan, and the bezafibrate clinical trial was performed at each collaborating institution. The study participants were recruited from January 2014 to December 2015. Bezafibrate was purchased from Kissei Pharmaceutical Co., Ltd., Nagano, Japan.

### 2.4 Patients

We originally recruited patients with CPT-2, VLCAD, TFP, MAD, and CACT deficiencies whose diagnoses were genetically or enzymologically confirmed. The target patient age was from 3 to 60 years old. Patients with renal disorder (creatinine >1.5 mg/dL), with liver or heart failure requiring medication, with a gallstone, or taking hydroxymethylglutaryl-CoA reductase inhibitor (hypolipidemic drug) and female patients who were pregnant or expecting pregnancy were excluded from the study. Patients who manifested episodic attacks with myopathic symptoms, such as general fatigue, severe myalgia, muscular weakness, or myoglobinuria twice or more for six months, or those with such episodes 4 times or more in a year and had high creatine kinase (CK) (over two-fold elevation of normal range) during episodes of myopathic symptoms were enrolled in this study. Ultimately, 8 patients (6 with VLCAD and 2 with CPT-2 deficiencies) were registered from February 2014 to May 2015. Clinical data of the 8 patients are shown in Table 1.

**Table 1:** Clinical features and genotypes of the patients before enrollment.

	VLCADD-1	VLCADD-2	VLCADD-3	VLCADD-4	VLCADD-5	VLCADD-6	CPT2D-1	CPT2D-2
Age	26 y	7 y	25 y	6 y	6 y	22 y	9 y	5 y
Sex	F	F	M	F	M	F	F	F
Diagnosis	VLCADD	VLCADD	VLCADD	VLCADD	VLCADD	VLCADD	CPT-2D	CPT-2D
Mutation	F113*	A333fs	Untested	R229X	L243F	E285G	R51G	F383Y
	K382Q	R450H	Untested	K382Q	V547 M	V400 M	E174K	R477W
Onset age	1.5 y	4.11 y	5 mo	Around 1 y	3 y	Around 13 y	1.3 y	3.7 y
Diagnosis age	5 y	5 y	13 y	0 mo	3 y	22 y	3 y	8 mo
Body weight (kg)	56	24	58	20	21	47	35	17
Clinical features	Myalgia or fatigue	Myalgia	Myalgia or fatigue	Myalgia or fatigue	Myalgia or rhabdomyolysis	Myalgia or rhabdomyolysis	Myalgia	Rhabdomyolysis or hyper CK
Frequency of								
Severe attacks	20/year	3/year	0	Several times/year	1–2/year	5/year	Several times/year	1/year
Moderate attacks	50–60/year	4/year	0	12/year	0	7/year	Uncountable	0
Mild attacks	Almost every day	6/year	2/year	Uncountable	0	12/year	Uncountable	0
Treatments								
Carnitine (mg/day)	750 mg	None	400–600 mg	almost none	None	1800 mg	1000 mg	900–1800 mg
MCT oil/milk	None	None	Yes	None	Yes	None	Yes	None
Restriction of activity	Prolonged walk and standing	PE, exercise, and excursion	Airing	Unclear	None	None	None	None
Responsiveness of bezafibrate *in vitro*	Good	Good	Untested	Good	Good	Good	Good	Untested
CK baseline (IU/L)	1933 ± 1220	180 ± 104	768 ± 612	1112 ± 1253	81 ± 13	590 ± 660	1201 ± 20	308 ± 169
C14:1 baseline (μM)	10.41 ± 4.64	1.18 ± 0.60	3.27 ± 4.05	2.98 ± 0.88	1.37 ± 1.77	1.36 ± 0.85		
C16 + C18:1 baseline (μM)							8.52 ± 5.40	6.94 ± 5.70

y, year; mo, month; M, male; F, female; VLCADD, very long-chain acyl-CoA dehydrogenase deficiency; CPT-2D, carnitine palmitoyltransferase-2 deficiency; PE, physical education. Frequency of attacks and treatments were provided in the year prior to enrolment. Responsiveness of bezafibrate *in vitro* was evaluated using the *in vitro* probe acylcarnitine assay [8].

Patient 1 (VLCADD-1) had a VLCAD deficiency and was a twenty-six-year-old female who developed tachypnea and metabolic acidosis soon after birth. At 1.5 years of age, she had an episode of vomiting, syncope, and seizure accompanied by an increased blood CK level and non-ketotic dicarboxylic aciduria following an infection. Thereafter, she frequently suffered from vomiting and elevated CK levels during infectious diseases. She was diagnosed with VLCAD deficiency at 5 years old by enzyme analysis using fibroblasts. Gene analysis identified mutations of p.F113*/p.K382Q in *ACADVL*. She suffered from either myalgia or general fatigue almost every day, which sometimes lead to hospitalization due to the inability to move, before the enrollment of this study.

VLCADD-2 was a seven-year-old girl who was diagnosed with VLCAD deficiency when she developed rhabdomyolysis associated with acute enteritis at 5 years old. She had compound heterozygous mutations of p.A333fs/p.R450H in *ACADVL*. She exhibited gait difficulty due to myalgia once or twice a month after diagnosis and presented with occasional high CK levels (over 10,000 IU/L) or mild liver dysfunction even in asymptomatic periods.

VLCADD-3 was a twenty-five-year old male. Although he exhibited hypoglycemia, seizure, and fatty liver at 5 months of age, he could not be diagnosed. He was hospitalized due to a high CK level triggered by an asthma attack at 5 years old, and at 12 years old, he frequently exhibited general fatigue or an elevated CK level. He was diagnosed with VLCAD deficiency based on the results of blood acylcarnitine (AC) analysis and an *in vitro* probe assay [19]. He was aware of mild myalgia and fatigue several times a month but lived normally, even with high CK levels, into adulthood.

VLCADD-4 was a six-year-old girl diagnosed with VLCAD deficiency by familial screening soon after birth because her mother was also affected with VLCAD deficiency, and her father was a heterozygous carrier. She had compound heterozygous mutations of p.R229*/p.K382Q in *ACADVL*. Although she was temporarily asymptomatic after diagnosis, she had been hospitalized for myalgia and fatigue two or three times a year since her first birthday. When she became more active in nursery school, her myopathic symptoms occurred more frequently. However, the frequency of her myopathic attacks was unknown. As her mother was well aware of the symptoms of VLCAD deficiency, the patient was monitored at home during muscle symptom episodes.

VLCADD-5 was a six-year-old boy who was diagnosed with VLCAD deficiency at 3 years of age. His initial symptoms were hypoglycemia, seizure, and high CK levels. Genetic analysis showed compound heterozygous mutations of p.L243F/p.V547 M in *ACADVL*. He suffered recurring bouts of rhabdomyolysis approximately once a month during l-carnitine treatment. However, his attacks almost disappeared after ceasing l-carnitine supplementation.

VLCADD-6 was a 22-year-old female. Since she was a junior high school student, myalgia or muscle weakness occurred several times a year after athletic festivals or climbing mountains. At 22 years old, she was hospitalized due to manifestations of rhabdomyolysis such as myoglobinuria and a high CK level (>100,000 IU/L) and was consequently diagnosed with VLCAD deficiency by muscle biopsy and Western blot analysis, which identified p.E285G/p.V400 M mutations. Although she received lifestyle guidance such as frequent intake of oral supplementation, her bouts of rhabdomyolysis occasionally recurred even following mild exercise [20].

CPT2D-1 was a nine-year-old girl with CPT-2 deficiency. Her younger brother also had CPT-2 deficiency (but was not enrolled in this study). She was hospitalized due to unconsciousness and convulsion at 15 months of age. She was suspected to have Reye-like syndrome accompanied by hypoglycemia and liver dysfunction. Since analyses of her blood AC and urinary organic acid showed elevations in long-chain ACs (particularly, C16 and C18:1) and hypoketotic dicarboxylic aciduria, respectively, CPT-2 or CACT deficiency was strongly suspected. Subsequently, her diagnosis of CPT-2 deficiency was confirmed by enzyme assay using fibroblasts. Genetic testing showed compound heterozygous mutations of p.R51G/p.E174K in *CPT2*. Before enrollment in this study, she occasionally suffered from myalgia triggered by fever or exercise. However, because she did not visit a hospital after experiencing muscle symptoms, it was not possible to accurately assess the frequency of her myopathic attacks.

CPT2D-2 was a five-year-old girl who was suspected of having CPT-2 deficiency by a pilot study of expanded newborn screening. Her diagnosis was confirmed by genetic analysis showing compound heterozygous mutations of p.F383Y/p.R477W at 8 months of age. Although she experienced normal development in infancy, her first metabolic attack of rhabdomyolysis with elevated CK levels (50,999 IU/L) occurred at 3 years and 7 months old. Thereafter, she suffered repeated bouts of rhabdomyolysis approximately twice a year following hyperpyrexia or exercise. However, she did not complain of muscle pain during the episodes.

### 2.5 Protocol

**Fig. 1:**
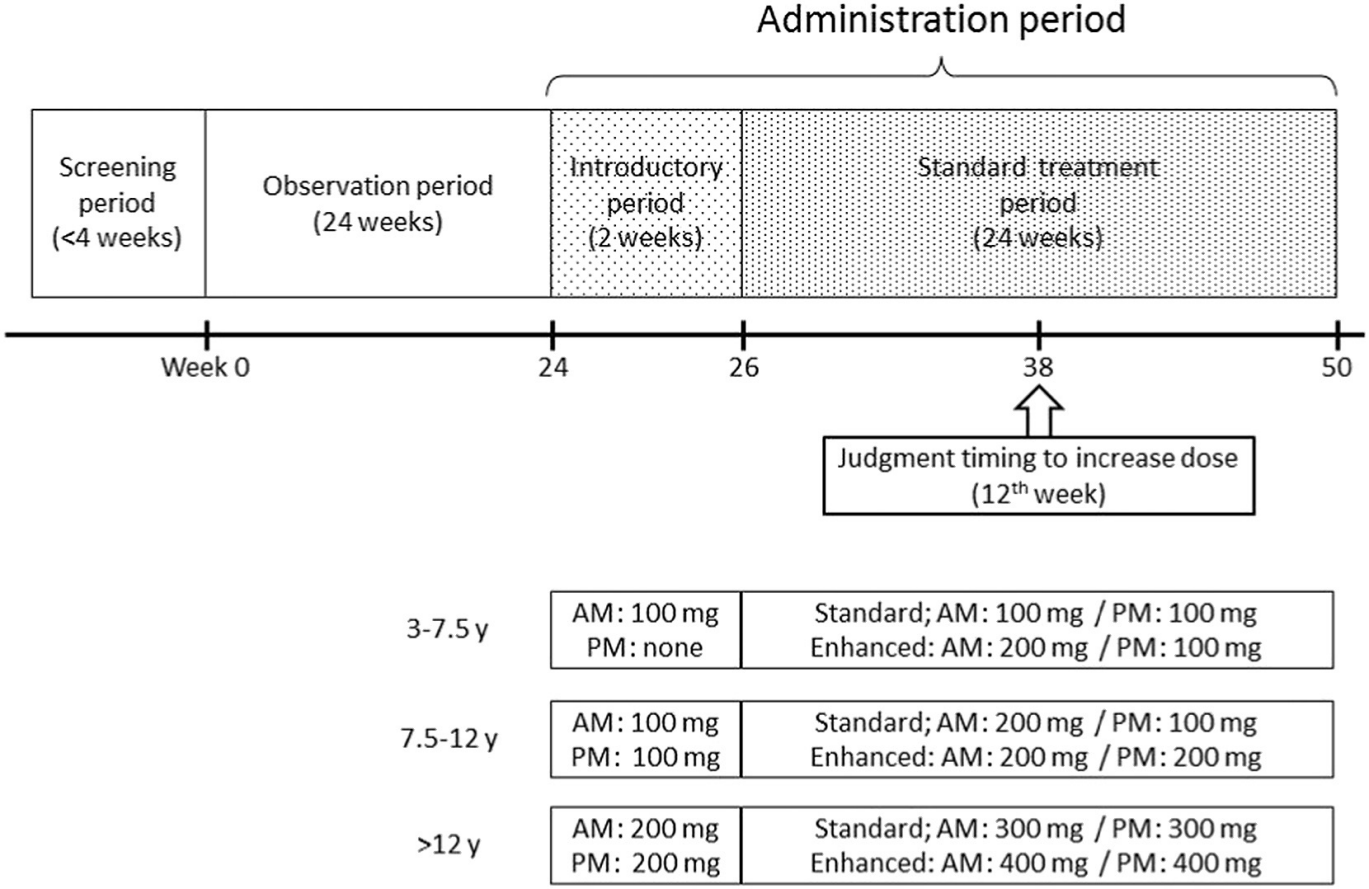
Study protocol. If patients were classified as having a severe clinical disease course such as continued frequent myopathic attacks at the 38th week (12 weeks after starting standard treatment), then enhanced-dose bezafibrate was permissible.

As shown in Fig. 1, this study was composed of 3 periods: 1) a screening period for 4 weeks; 2) an observation period for 24 weeks; and 3) an administration period for 26 weeks. The administration period was divided into a 2-week introductory period and a 24-week standard treatment period. During the screening period, serum levels of ACs and CK were measured three times over a 3-day interval during asymptomatic conditions. Average AC and CK levels were provided as a baseline that was used to determine the efficacy of bezafibrate and whether participants were experiencing a myopathic attack. Furthermore, each patient's quality of life (QOL) was also analyzed using a 36-Item Short Form Health Survey (SF-36) [21] during the same period. The observation period immediately followed determination of baseline AC and CK levels, irrespective of the screening period time-frame. During the 24-week observation period, the frequency of myopathic attacks, serum CK and AC levels and degree of myalgia using a visual analog scale (VAS) during the metabolic attacks were investigated. The elevation of VAS score implicated pain aggravation. After the observation period, low-dose bezafibrate was administered for 2 weeks during the introductory period. Bezafibrate efficacy was not investigated during this period. The bezafibrate was administered as tablets regardless of patient age because the tablets use a slow-release system.

The dose of bezafibrate was determined based on age (100, 200, and 400 mg/day for patients aged 3–7.5, 7.5–12, and >12 years, respectively). Subsequently, treatment with standard-dose bezafibrate (200, 300, and 600 mg/day for patients aged 3–7.5, 7.5–12, and >12 years, respectively) was initiated and continued for 24 weeks. If standard-dose bezafibrate did not reduce the frequency of myopathic attacks by the 12th week of treatment, then an enhanced treatment period of high-dose bezafibrate (300, 400, and 800 mg/day for patients aged 3–7.5, 7.5–12, and >12 years, respectively) could be selected from the 12th to 24th week. The frequency of myopathic attacks and associated serum levels of CK and AC were investigated during the standard or enhanced treatment period as well as during the observation period. Patient QOL was re-evaluated at the end of the administration period using the SF-36. The drug compliance was calculated as (prescription number – remainder number)/prescription number. There were regular blood examinations and laboratory data such as cholesterol and transaminase levels collected once every two to four weeks during the treatment period to monitor adverse effects. CK and AC levels were not examined in routine assessments.

### 2.6 Endpoint

We compared the frequency of myopathic attacks before and after bezafibrate treatment as a primary endpoint. A myopathic attack was defined as clinical features with a moderate or greater degree of muscle symptoms such as general or extremity fatigue, muscle stiffness or weakness, myalgia, or myoglobinuria that accompanied an elevated CK level. In infants, poor activity and ill temper were considered myopathic symptoms. An elevated CK level was defined as a >5-fold increase over baseline. While a moderate degree of muscle symptoms indicated that patients experienced difficulties in performing routine functions such as eating, dressing, toilet activity, and bathing due to muscle symptoms, a severe degree demonstrated that patients could not perform routine functions at all. A mild degree of muscle symptoms was defined as being able to perform routine activity despite myalgia and fatigue and was not considered a myopathic attack.

Secondary endpoints were changes in CK, AC, and VAS values during the myopathic attacks and QOL before and after bezafibrate treatment. Serum AC levels specific to each FAOD, for example, C14:1 in VLCAD deficiency and C16 + C18:1 in CPT-2 deficiency, were investigated. The VAS represented the degree of myalgia based on a pain scale of 0 to 100. QOL was quantitated using the SF-36 questionnaire, which consisted of eight components such as physical functioning (PF), role limitation due to physical problems (role physical; RP), bodily pain (BP), general health perception (GHP), vitality (VT), social functioning (SF), role limitation due to emotional problems (role emotional; RE), and mental health (MH).

### 2.7 Data statistics and analysis

Although registration of approximately 58 patients was necessary to obtain 80% statistical power to detect a difference between before (the null average frequency of myopathic attacks = 3 per 24 weeks [12]) and after bezafibrate treatment (the expected average frequency of myopathic attacks = 2 per 24 weeks) based on a Poisson distribution with two-sided α of 5%, only eight patients could participate in our study. Therefore, we simply compared changes in individual endpoints before and after bezafibrate treatment. Only QOL data were analyzed using a paired *t*-test for comparisons of the two periods using SAS version 9.4 (SAS Institute Inc., Cary, NC, USA). Data were expressed as the mean ± standard deviation, or median (minimum to maximum) as appropriate.

## 3 Results

All patients (6 with VLCAD and 2 with CPT-2 deficiencies), including 2 males and 6 females, completed this study. There were no dropouts. The median age was 6.9 (5.8 to 26.4) years. Age brackets were assigned, and four patients were aged 3–7.5 years old, one was 7.5–12 years old, and the remaining 3 were >12 years old. Only CPT2D-1 was treated with enhanced-dose bezafibrate. Because the metabolic attacks of CPT2D-1 were increased after bezafibrate treatment, 400 mg of bezafibrate, which was an enhanced dose for patients aged 7.5–12 years, was administered to CPT2D-1 at the 38th week for 12 weeks following the standard treatment. Adverse drug reactions were not observed in any cases during the administration period. All patients strictly complied with this protocol, namely by immediately visiting a hospital in the event of metabolic attack or maintaining a consistent lifestyle before and after treatment, except for CPT2D-1, who joined a school athletic festival during the administration period. All patients had >80% of drug compliance (range 83.1 to 100%, median 98.0%).

### 3.1 Primary endpoint

The number of myopathic attacks decreased in three patients (VLCADD-2, VLCADD-4, and VLCADD-6), increased in three patients (VLCADD-1, CPT2D-1, and VLCADD-5), and did not change in two patients (VLCADD-3 and CPT2D-2) (Table 2). In the group with decreased attacks, VLCADD-4 and VLCADD-6 had no myopathic attacks during the administration period, although they had attacks twice during the observation period. The number of myopathic attacks for VLCADD-2 decreased from 5 times before treatment to 4 times after treatment. The number of myopathic attacks for VLCADD-1, VLCADD-5 and CPT2D-1 increased from 4 to 5, 1 to 2, and 1 to 4, respectively. Although CPT2D-1 was treated with enhanced-dose bezafibrate starting at the 38th week, the frequency of his myopathic episodes did not decrease during the enhanced treatment period. VLCADD-3 and CPT2D-2 had no attacks before or after bezafibrate treatment.

**Table 2:** Comparison of the number of myopathic attacks.

	Before	After
VLCADD-1	4	5
VLCADD-2	5	4
VLCADD-3	0	0
VLCADD-4	2	0
VLCADD-5	1	2
VLCADD-6	2	0
CPT2D-1	1	4
CPT2D-2	0	0

We evaluated the extent of myopathic symptoms. All myopathic attacks were considered to be of a moderate degree before and after treatment, except for CPT2D-1. CPTD-1 demonstrated severe myopathic symptoms during both the observation and treatment periods, which indicated that the degree of myopathic symptoms remained unchanged after the treatment.

### 3.2 Secondary endpoints

#### 3.2.1 Serum CK level during myopathic attacks

The average CK level during attacks decreased in two patients (VLCADD-1 and CPT2D-1), increased in two (VLCADD-2 and VLCADD-5) and was not evaluated in 4 (VLCADD-3, VLCADD-4, VLCADD-6, and CPT2D-2) (Table 3). The number of VLCADD-1’s attacks increased from 4 to 5 during the treatment period, and the average CK level during attacks marginally decreased from 23,905 to 23,235 IU/L; however, overall CK levels during each attack largely varied (Table 8). Although the number of attacks increased from 1 to 4 times in CPT2D-1, the average CK level was reduced from 23,403 to 1269 IU/L. In contrast, despite this reduction in the number of attacks, the average CK level increased from 8714 to 9599 IU/L in VLCADD-2. Additionally, in VLCADD-5, the frequency of attacks increased, and the average CK level was elevated from 594 to 2783 IU/L after bezafibrate treatment. Changes in the CK level during attacks in the other 4 patients (VLCADD-3, VLCADD-4, VLCADD-6, and CPT2D-2) were indeterminable because they did not experience any attacks before or after treatment. The severity of myopathic symptoms was not correlated with CK levels (Table 8). During the severe attacks the CK levels were 23,403 IU/L before the treatment and 1248 IU/L after treatment in CPT2D-1.

**Table 3:** Comparison of the CK levels during myopathic attacks.

	Before	After	(Difference)
VLCADD-1	23,905 ± 23,726	23,235 ± 11,505	(−670)
VLCADD-2	8714 ± 1578	9599 ± 7407	(885)
VLCADD-3	–	–	
VLCADD-4	8943 ± 2940	–	
VLCADD-5	594	2783 ± 1559	(2189)
VLCADD-6	11,325 ± 11,418	–	
CPT2D-1	23,403	1269 ± 313	(−22,134)
CPT2D-2	–	–	

#### 3.2.2 AC levels during myopathic attacks

In VLCAD deficiency, the C14:1 level during myopathic attacks increased in two patients (VLCADD-1 and VLCADD-2) after treatment, whereas this level was reduced in VLCADD-5. Conversely, C16 + C18:1 was reduced in CPT2D-1 (Table 4). Similar to the CK results, the changes in AC levels during myopathic attacks before and after treatment could not be investigated in 4 patients because they did not experience any attacks during the observation or administration periods.

**Table 4:** Comparison of the AC levels during myopathic attacks.

	Before	After	(Difference)
C14:1 (μM)			
VLCADD-1	3.85 ± 2.75	6.39 ± 6.96	(2.54)
VLCADD-2	9.44 ± 4.01	17.33 ± 2.89	(7.89)
VLCADD-3	–	–	–
VLCADD-4	7.26 ± 6.37	–	–
VLCADD-5	7.06	6.28 ± 3.15	(−0.79)
VLCADD-6	0.570 ± 0.014	–	–
C16 + C18:1 (μM)			
CPT2D-1	38.39	7.73 ± 2.51	(−30.67)
CPT2D-2	–	–	–

Although both the frequency of myopathic attacks and the CK level increased in VLCADD-5 during the administration period, his average C14:1 level during myopathic attacks decreased from 7.06 to 6.275 μM. In VLCADD-1, whose CK level was decreased, the average C14:1 level increased from 3.85 to 6.392 μM, and the number of attacks also increased after treatment. In VLCADD-2, the number of attacks was reduced, but the C14:1 level increased from 9.44 to 17.328 μM, which coincided with a mild elevation in CK. In contrast, average C16 + C18:1 level in CPT2D-1 reduced from 38.39 to 7.73 μM with a concomitant decrease in CK level despite the increased number of attacks.

#### 3.2.3 VAS during myopathic attacks

Differences between VAS scores before and after treatment could be investigated in only three patients (VLCADD-1, VLCADD-2, and CPT2D-1) because VAS was not assessed in VLCADD-5 during the observation period, and myopathic attacks were not observed before or after bezafibrate treatment in other cases. VAS scores increased in VLCADD-1 (64.5 to 77.0), whereas scores decreased in VLCADD-2 (49.4 to 34.5) and CPT2D-1 (75.0 to 56.5) during the administration period (Table 5). The elevated VAS score indicated pain aggravation.

**Table 5:** Comparison of the VAS scores during myopathic attacks.

	Before	After	(Difference)
VLCADD-1	64.5 ± 15.0	77.0 ± 1.9	(12.5)
VLCADD-2	49.4 ± 11.7	34.5 ± 17.7	(−14.9)
VLCADD-3	–	–	–
VLCADD-4	43.5 ± 2.1	–	–
VLCADD-5	–	64.5 ± 21.9	–
VLCADD-6	48.0 ± 39.6	–	–
CPT2D-1	75.0	56.5 ± 27.0	(−18.5)
CPT2D-2	–	–	–

Elevation of VAS score indicates pain aggravation.

#### 3.2.4 QOL using the SF-36 questionnaire

**Fig. 2:**
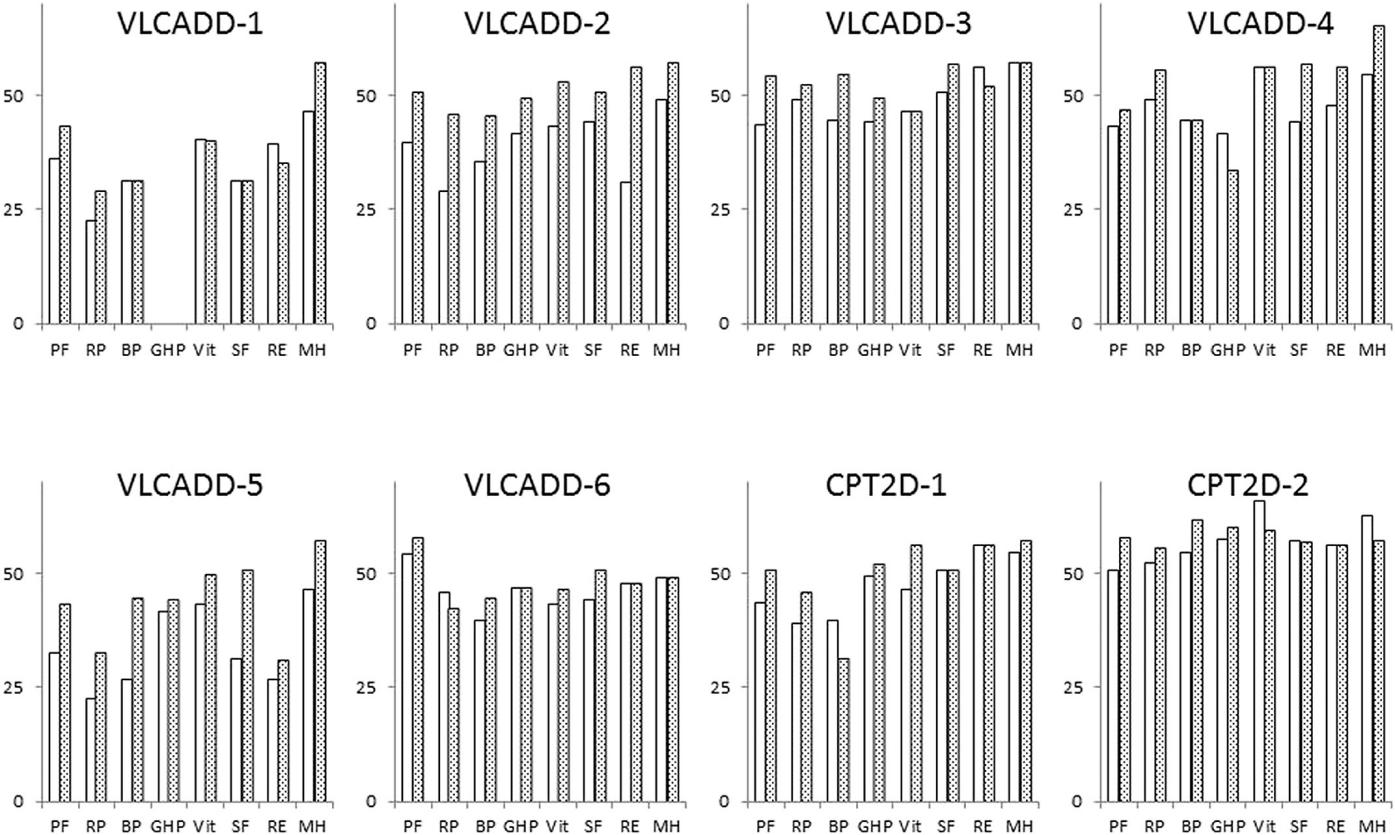
Quality of life evaluation in each patient. White and gray bars indicate before and after the treatment, respectively. PF, physical functioning; RP, role limitation due to physical problems; BP, bodily pain; GHP, general health perception; VT, vitality; SF, social functioning; RE, role limitation due to emotional problems; MH, mental health.

The QOL questionnaire results were obtained at the end of screening period (before treatment) and treatment period (after treatment). The elevation of QOL score indicated improvement of QOL. All components of QOL were evaluated except for GHP in VLCADD-1. The average total QOL score was significantly elevated from 351.6 ± 69.1 to 390.0 ± 58.7 after bezafibrate treatment (P = 0.01) (Table 6). There were no patients whose total QOL score decreased. All QOL components were improved in VLCADD-2 and VLCADD-5, while several components were improved in the other patients (Fig. 2).

**Table 6:** Comparison of the total QOL scores using the SF-36 before and after treatment.

	Before	After	(Difference)
VLCADD-1	247.4	267.8	(20.4)
VLCADD-2	313.5	408.4	(94.9)
VLCADD-3	392	423.4	(31.4)
VLCADD-4	381.3	415.5	(34.2)
VLCADD-5	271.5	353.5	(82)
VLCADD-6	371	385.8	(14.8)
CPT2D-1	379.7	400.2	(20.5)
CPT2D-2	456.7	465.2	(8.5)
Average	351.6	390.0	P = .01

Elevation of QOL score indicates improvement of QOL.

When analyzing each QOL component, three components, PF, RP, and SF, significantly improved after treatment, such that the PF, RP, and SF scores were elevated from 43.0 to 50.6 (P < 0.01), 38.7 to 44.9 (P = 0.02), and 44.1 to 50.6 (P = 0.03), respectively (Table 7). Scores of the other five components were also elevated but not significantly.

**Table 7:** QOL results from the SF-36 administered before and after treatment.

	Before	After	p value
Physical functioning (n = 8)	43.0	50.6	<0.01
Role physical (n = 8)	38.7	44.9	0.02
Bodily pain (n = 8)	39.7	44.9	0.11
General health perceptions (n = 7)	46.1	48.0	0.36
Vitality (n = 8)	48.2	51.0	0.19
Social functioning (n = 8)	44.1	50.6	0.03
Role emotional (n = 8)	45.1	48.8	0.32
Mental health (n = 8)	52.5	57.2	0.07

## 4 Discussion

Our study was an open-label prospective clinical trial of bezafibrate treatment in patients with VLCAD and CPT-2 deficiencies. Although our data analysis involved simple comparisons and was not statistically analyzed due to a small study population, the frequency of myopathic attacks decreased in some patients after bezafibrate treatment but increased in others. Similarly, CK, AC, and VAS values during attacks varied between individuals. Because there were a few flaws in our study design, the efficacy of bezafibrate on the frequency of myopathic attacks and CK, AC, and VAS values during attacks remained undetermined. However, our results suggested that bezafibrate improved the QOL of patients with FAODs.

To investigate the frequency of myopathic attacks as a primary endpoint, we attempted to recruit patients who continued to have intermittent metabolic attacks despite standard treatments such as diet therapy and restriction of over exercise. However, only 8 patients participated in this trial, which was less than originally expected and insufficient to perform statistical analyses, even if there was a clear trend in some endpoints. Nevertheless, some QOL components were significantly improved despite the small study population. Additionally, *in vitro* responsiveness to bezafibrate was not measured in all participants, and this was one of major problems in our study. Although six participants were responders *in vitro*, the number of attacks was not decreased in VLCADD-1, VLCADD-5, and CPT2D-1. This difference might be due to inadequate design of this study, and the *in vitro* responsiveness of all patients should have been inspected before enrollment in the trial.

In our study, “myopathic attack” was defined as a 5-fold increase over baseline CK levels, which was ultimately one of the limitations of this study. This cutoff was derived from criteria of rhabdomyolysis in which the CK level is elevated 5- to 10-fold higher than normal level [22,23]. However, although the baseline level of CK was obtained in “the asymptomatic condition”, it was higher than the CK level in the “real” stable condition in some patients (data not shown). This complicated obtaining data that met the definition of a myopathic attack. Indeed, even though the patients visited the hospital with myalgia and general fatigue, such episodes are sometimes not regarded as a ‘myopathic attack’ due to insufficient increases in the CK level. Therefore, the frequency of myopathic attacks observed during the observation and administration periods was lower than that before initiation of this study. Moreover, such episodes might also be influenced by a time lag in the elevation of serum CK levels [24]. The elevation of CK might be delayed following the manifestation of patient symptoms such as myalgia and fatigue. Therefore, it is likely that the number of attacks and CK levels during attacks were underestimated. Although we requested that all participants visited an attending physician as soon as possible upon first muscle symptoms, sampling time might be different for each individual due to various factors, such as distance to the hospital. This also might have contributed to the variability of CK levels and makes it difficult to interpret our results. Furthermore, it is possible that child patients tended to be more active after bezafibrate administration, since muscle weakness and myalgia became milder than before administration. We requested all participants to keep their daily life and activity consistent before and after the treatment. However, the patients might have been more physically active, which was noted by the attending physicians. This concept also may be one explanation for why the number of myopathic attacks, CK, ACs, and VAS did not decrease markedly. We could not validate the extent of the change in physical activity of the patients because this was not a study endpoint. Therefore, future studies should track physical activity using a diary because it is more informative than biochemical markers in this kind of trial.

This study protocol determined CK, AC, and VAS values exclusively at the time of myopathic attacks. As a result, we could not obtain considerable quantities of data and ultimately excluded 4 patients. There were two patients who did not experience attacks after bezafibrate administration. This finding indicated that our results on the changes in CK, AC, and VAS values during attacks did not reflect those 2 cases who were likely to be good responders to bezafibrate treatment. Additionally, the treatment period might have been too short to establish the efficacy of bezafibrate. We found that VLCADD-1 and CPT2D-1, who were considered non-responders in this study, became physically fit when they continued with bezafibrate therapy after our study.

The QOL scores were collected using the SF-36 for all participants regardless of the status of myopathic attacks. The three QOL components of PF, RP, and SF were significantly improved after bezafibrate treatment. The other five components also improved, but the changes were not statistically significant. Thus, it is difficult to conclude that bezafibrate improved patient QOL only using these results. However, we considered that it was very meaningful to have statistical significance for 3 of the 8 QOL components with our very small participant population.

SF-36 has been used in various studies and is a reliable questionnaire that evaluates both physical and mental functions [21,25,26]. Bonnefont et al. previously reported that SF-36 values of patients with CPT-2 deficiency improved after bezafibrate treatment [13]. Although our study and Bonnefont's report could be influenced by a placebo effect due to being an open-label study, myopathic symptoms could not be objectively estimated even using the biomarkers CK and AC. This is because the VAS, which quantifies subjective pain, does not correlate with the CK or AC levels, as shown in Table 8. Hence, although estimating QOL using SF-36 was subjective and a secondary endpoint, we believe that the QOL values are the most trustworthy results in this study. Even in CPT2D-1 and VLCADD-5, who showed no response to bezafibrate treatment regarding the frequency of myopathic attacks, almost all components of QOL were increased, suggesting that bezafibrate is effective for improving QOL. Additionally, more than half of the patients and attending physicians vaguely realized “some kind of bezafibrate efficacy” even though the frequency of myopathic attacks, degree of muscle symptoms, and biochemical markers such as CK levels did not significantly improve. If “some kind of efficacy” can be correctly investigated, bezafibrate will be a promising drug for FAODs.

**Table 8:** CK level, acylcarnitines, and VAS score for each attack.

	Attack number	Observation period					Administration period				
		1	2	3	4	5	1	2	3	4	5
VLCADD-1	CK	10,274	16,292	59,220	9837		35,493	33,855	11,050	12,374	23,405
	C14:1	7.70	3.39	1.16	3.16		1.69	5.53	2.68	18.59	3.47
	VAS	56	58	87	57		78	77	79	74	77

VLCADD-2	CK	8547	8622	6231	10,086	10,085	3326	4025	18,987	12,061	
	C14:1	11.97	8.55	3.45	9.19	14.04	14.32	16.00	21.05	17.94	
	VAS	36	52	67	50	42	24	48	51	15	

VLCADD-4	CK	11,023	6864								
	C14:1	11.76	2.75								
	VAS	45	42								

VLCADD-5	CK	594					3886	1681			
	C14:1	7.06					4.05	8.50			
	VAS	–					80	49			

VLCADD-6	CK	3251	19,399								
	C14:1	0.56	0.58								
	VAS	20	76								

CPT2D-1	CK	23403^a^					1695	1248^a^	1192	941	
	C16 + C18:1	38.39^a^					9.79	5.55^a^	5.55	10.01	
	VAS	75^a^					60	18^a^	80	68	

a Severe myopathic symptoms.

Lastly, we did not experience any bezafibrate-related adverse events or unexpected incidents during the entire period of this study, even though our data contained a patient who received high dose of bezafibrate (400 mg/day for a 9-year-old patient). For decades, bezafibrate has been used to treat many patients with hyperlipidemia, and in this study, we confirmed its safety in patients with FAODs.

## 5 Conclusion

Our study failed to demonstrate the evident efficacy of bezafibrate treatment on the frequency of myopathic attacks, CK and AC levels, or VAS, but this study did not negate its effectiveness either. However, although bezafibrate efficacy remains controversial, our findings support one of its benefits in improving the QOL of patients with FAODs. Further studies with additional clinical trials are essential to elucidate the efficacy of bezafibrate for patients with FAODs.

## Sources of funding

This study was supported by “The multicenter clinical trial of the efficacy and safety of bezafibrate for mitochondrial fatty acid β-oxidation disorders (Grant Number, 16lk0103005h0005)” from the Japan Agency for Medical Research and Development (AMED).

## Conflicts of interest

The authors have no conflicts of interest (COIs) to declare regarding the publication of this manuscript.

## Contributions

K. Yamada and H. Shiraishi equally contributed to the submission of this manuscript, namely, by participating in the study conception and design, figure acquisition, and data interpretation and by drafting, revising, and finalizing the manuscript. Additionally, H. Shiraishi, S. Yamaguchi, and N. Sato extensively organized the writing of this manuscript and performed and designed this trial as principal investigators. N. Asahina first conceived this trial and participated in the study by acquiring the funding source, analyzing and interpreting the data, and revising the manuscript. S. Yokoshiki, T. Miyakoshi, T. Isoe, and H. Hayashi contributed to the registration, design, governance, proceeding, and intermediacy of this trial as the main clerical organizers of this study. K. Ono and K. Oba, who are trial statisticians, participated in the study conception and design and data analysis and interpretation. E. Oki, M. Ishige, T. Fukao, Y. Hamada, N. Sakai, F. Ochi, A. Watanabe, S. Kawakami, K. Kuzume, K. Watanabe, K. Sameshima, K. Nakamagoe, and A. Tamaoka contributed to the design of this trial and provided clinical data, suggestions, and data interpretation as attending doctors and investigators.
